# Neurogenic Heterotopic Ossifications Recapitulate Hematopoietic Stem Cell Niche Development Within an Adult Osteogenic Muscle Environment

**DOI:** 10.3389/fcell.2021.611842

**Published:** 2021-03-05

**Authors:** Dorothée Girard, Frédéric Torossian, Estelle Oberlin, Kylie A. Alexander, Jules Gueguen, Hsu-Wen Tseng, François Genêt, Jean-Jacques Lataillade, Marjorie Salga, Jean-Pierre Levesque, Marie-Caroline Le Bousse-Kerdilès, Sébastien Banzet

**Affiliations:** ^1^INSERM UMRS-MD 1197, Institut de Recherche Biomédicale des Armées (IRBA), Clamart, France; ^2^INSERM UMRS-MD 1197, Université Paris-Saclay, Hôpital Paul Brousse, Villejuif, France; ^3^Mater Research Institute—The University of Queensland, Woolloongabba, QLD, Australia; ^4^INSERM U1179, Université de Versailles Saint-Quentin-en-Yvelines (UVSQ), Versailles, France

**Keywords:** neurogenic heterotopic ossifications, ectopic hematopoietic niche, muscle environment, inflammation, macrophages

## Abstract

Hematopoiesis and bone interact in various developmental and pathological processes. Neurogenic heterotopic ossifications (NHO) are the formation of ectopic hematopoietic bones in peri-articular muscles that develop following severe lesions of the central nervous system such as traumatic cerebral or spinal injuries or strokes. This review will focus on the hematopoietic facet of NHO. The characterization of NHO demonstrates the presence of hematopoietic marrow in which quiescent hematopoietic stem cells (HSC) are maintained by a functional stromal microenvironment, thus documenting that NHOs are neo-formed ectopic HSC niches. Similarly to adult bone marrow, the NHO permissive environment supports HSC maintenance, proliferation and differentiation through bidirectional signaling with mesenchymal stromal cells and endothelial cells, involving cell adhesion molecules, membrane-bound growth factors, hormones, and secreted matrix proteins. The participation of the nervous system, macrophages and inflammatory cytokines including oncostatin M and transforming growth factor (TGF)-β in this process, reveals how neural circuitry fine-tunes the inflammatory response to generate hematopoietic bones in injured muscles. The localization of NHOs in the peri-articular muscle environment also suggests a role of muscle mesenchymal cells and bone metabolism in development of hematopoiesis in adults. Little is known about the establishment of bone marrow niches and the regulation of HSC cycling during fetal development. Similarities between NHO and development of fetal bones make NHOs an interesting model to study the establishment of bone marrow hematopoiesis during development. Conversely, identification of stage-specific factors that specify HSC developmental state during fetal bone development will give more mechanistic insights into NHO.

## Introduction

**FIGURE 1:**
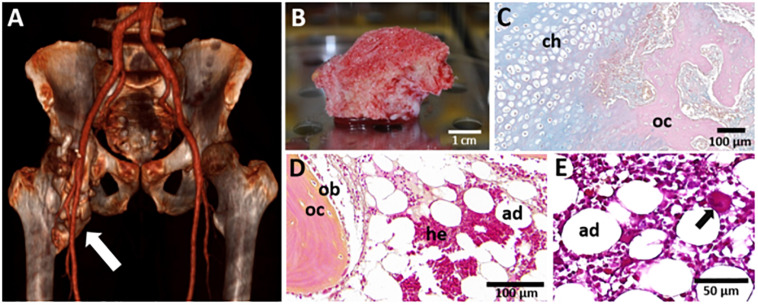
Macroscopic and microscopic views of NHO. **(A)** 3D scan of a NHO (white arrow) located in the right hip of a patient; **(B)** NHO biopsy resected during surgery displaying vascularized medullary cavities (scale bar 1 cm); **(C)** Alcian Blue-Nuclear Red staining of an NHO section showing the endochondral ossification process with the presence of chondrocytes (ch) and osteocytes (oc) (scale bar 100 μm). Hematoxylin-Phloxine-Safran staining of an NHO section showing the presence of **(D)** osteoblasts (ob), osteocytes (oc), and medullar cavities displaying adipocytes (ad), hematopoietic cells (he) (scale bar 100 μm) and **(E)** a megakaryocyte (black arrow) (scale bar 50 μm).

Heterotopic ossification (HO) is an abnormal development of bone tissue within soft tissue. HO can be hereditary such as Fibrodysplasia Ossificans Progressiva (FOP) or acquired following traumatic injuries and burns (Meyers et al., 2019). Among acquired HO, neurogenic heterotopic ossifications (NHO) are pathological formations of ectopic bones in peri-articular muscles following severe central nervous system (CNS) lesions such as traumatic brain injuries (TBI), stroke, cerebral anoxia or spinal cord injuries (SCI) (Genêt et al., 2011). NHOs develop near or around the hip, knee, elbow, and shoulder causing decreased range of motion which can extend to complete joint ankylosis, severe pain, nerve and vessel compression as it grows (Brady et al., 2018; Figure 1A). Large NHOs hamper functional recovery after CNS lesion and interfere with the rehabilitation program delaying potential neurological recovery (van Kampen et al., 2011). NHO incidence ranges from 10 to 23% in TBI patients, 10–53% in SCI patients (Garland, 1988; Brady et al., 2018) and up to 65% following blast injuries (Potter et al., 2007; Forsberg et al., 2009). The only curative option is surgical excision, but surgery remains challenging, especially when NHO entraps the affected joint, as well as proximal vessels and nerves.

Despite identifying NHOs in World War I injured soldiers (Dejerine and Ceillier, 1918), their pathogenesis is still poorly understood. Since most of the previous studies in SCI/TBI patients were retrospective, only a clinically relevant animal model could provide insights into the early events of NHO pathogenesis. Until recently there was no animal model that included the CNS lesions (Brady et al., 2018). Most models of heterotopic ossification (HO) were based on the activation of bone morphogenetic proteins (BMPs) pathway or a constitutively active ACVR1 receptor mutations as found in FOP, an extremely rare genetic disease caused by activating point mutations of the *ACVR1* gene encoding a type I BMP receptor (Kan et al., 2004; Shore et al., 2006; Chakkalakal et al., 2012). The relevance of these models to NHO is questionable because NHOs develop in a broad range of ethnicities in subjects otherwise genetically normal. To fill this knowledge gap, we established the first mouse model of NHO (Genêt et al., 2015). As NHO prevalence is high in multi-traumatic combat casualties (Forsberg et al., 2009; Citak et al., 2012), our model combines a SCI with muscle damage induced by an intramuscular injection of cardiotoxin. NHO only develops in the injured muscle when SCI is associated to the muscle injury, suggesting that it requires a dual insult (Alexander et al., 2020).

In NHO located near the hip, one of the most striking features is the presence of richly vascularized mature trabecular bones which contain medullary cavities filled with hematopoietic cells (HCs) (Davis et al., 2013; Torossian et al., 2017; Figure 1B). The pathological formation of an ectopic bone containing a hematopoietic bone marrow tissue in the adult is of foremost interest and the underlying mechanisms are yet to be fully elucidated. This review focuses on how animal models and studies performed with patient-derived cells can help further understand two key events of the establishment of ectopic hematopoiesis in NHO: the occurrence of a heterotopic ossification within skeletal muscle tissue, and the development of a functional hematopoiesis tissue within this heterotopic bone.

## Fibro-Adipogenic Progenitors and Altered Muscle Environment, Two Major Players in the Pathogenesis of NHO

It is currently admitted that NHOs are the result of an endochondral ossification process although intramembranous ossification has also been suggested (Cipriano et al., 2009; Cholok et al., 2018). Foley et al. have highlighted the presence of chondrocytes and a cartilaginous matrix on NHO biopsies and pinpointed different stages of NHO development including lymphocytic infiltration, fibro-proliferation, neovascularization, cartilage formation, and endochondral bone formation (Foley et al., 2018). A similar thick cartilaginous matrix displaying chondrocytes adjacent to cancellous bone and marrow is described in NHO 3–4 months after initial injury and becomes thinner at a later stage of NHO development named the “maturation” stage (Wang et al., 2018). Our group and others have evidenced the presence of hematopoietic sites associated with chondrocytes, osteoblasts/osteocytes, and adipocytes in mature trabecular bone in human NHO biopsies (Davis et al., 2013; Torossian et al., 2017; Figures 1C,D). Thus, the progressive formation of a cartilage intermediate maturing into a mineralized bone matrix associated with a vascularization network offers a suitable environment for the recruitment and homing of circulating HCs (Figure 1E; Chan et al., 2009; Kollet et al., 2012).

### Muscle Fibro-Adipogenic Progenitors: The Cells-of-Origin of NHO?

The development of heterotopic bones in muscles after severe CNS trauma raises interesting stem cell biology questions particularly regarding the cells-of-origin of NHO. Adult skeletal muscles contain two major types of progenitor cells participating in muscle regeneration. Myogenic satellite cells (SCs) are CD56 expressing stem cells located between the basal lamina and myofiber plasma membrane. To regenerate damaged myofibers, activated SCs proliferate, differentiate into myoblasts and fuse to form multinucleated myofibers with the support of macrophages, endothelial cells (ECs), fibroblasts and pericytes (see reviews: Collins et al., 2005; Bentzinger et al., 2013). On the other hand, fibro-adipogenic progenitors (FAPs) are interstitial mesenchymal stromal cells (MSCs) expressing platelet-derived growth factor receptor α (PDGFRα). FAPs provide growth factors and extracellular matrix to support SC proliferation and myogenic differentiation (see review: Joe et al., 2010; Wosczyna and Rando, 2018).

The cellular origin of NHO is still under investigation but numerous arguments incriminate FAPs rather than SCs. Both human SCs and FAPs show a capacity of osteoblastic differentiation *in vitro*, however, only PDGFRα^+^ FAPs maintained their osteogenic capacity in an *in vivo* ectopic bone model in immune-deficient mice (Oishi et al., 2013). In a burn injury/tenotomy mouse model, parabiosis experiments highlighted the involvement of circulating PDGFRα^+^ FAPs in the development of burn-induced HO in tendons (Loder et al., 2018). Another study combining parabiosis and a BMP-2-induced HO mouse model reported an abnormal accumulation PDGFRα^+^ FAPs associated with an *in vivo* osteogenic potential, although no circulating FAPs were detected (Eisner et al., 2020). In lineage-tracing experiments in which either SCs (via the endogenous *Pax7* gene promotor) or FAPs (via a *Prrx1* gene enhancer transgene) are specifically labeled, we find that following SCI, NHO are derived from *Prrx1* expressing FAPs, not from *Pax7* expressing SCs (Tseng et al., 2019).

The involvement of pericytes in HO remains debated (Matthews et al., 2016; Dey et al., 2017). Interestingly, a pericyte population expressing Glast was identified in a BMP-4-induced HO model with a subset co-expressing PDGFRα (Kan et al., 2013). Scleraxis (Scx)^+^ PDGFRα^+^ tenocytes are also involved in HO development in tendons using burn/tenotomy and BMP-induced mouse models and represent another interesting lead (Agarwal et al., 2017; Giordani et al., 2019). However, this model of burn-induced calcifying tendinopathy may be different from intramuscular NHO.

### Hypoxia and Inflammation as Drivers of NHO Development in Skeletal Muscles?

The molecular microenvironment of the muscle can dramatically affect the behavior and fate of SCs and FAPs (Malecova et al., 2018). A hypoxic microenvironment, mainly linked to inflammation and vascular damage, is an initiator and driver of ossification in acquired HO and FOP mouse models through the activation of Hypoxia Inducible Factor-1α (Agarwal et al., 2016; Wang et al., 2016). The local or systemic production of inflammatory mediators that stimulate the recruitment of MSCs, endothelial progenitors or other stem cells from the bone marrow and alter tissue repair have been proposed to provide a microenvironment/matrix supporting mineralization (Wang et al., 2004; Davis et al., 2013). Signaling molecules including BMPs and TGF-β could contribute in this altered microenvironment (Dey et al., 2017; Wang et al., 2018). Mononucleated phagocytes recruited in the injured muscle play a key role, as treatment with clodronate loaded liposomes, which deplete phagocytes *in vivo*, abolished NHO onset (Genêt et al., 2015). In contrast, polymorphonuclear (neutrophils) and polynucleated (osteoclasts) phagocytes are not necessary for NHO development (Genêt et al., 2015; Tseng et al., 2020). SCI exacerbates macrophage infiltration into injured muscles with increased and persistent expression of oncostatin M (OSM) (Torossian et al., 2017), a cytokine participating in both inflammation and hematopoiesis (Tanaka and Miyajima, 2003; Stawski and Trojanowska, 2019). The persistent OSM expression in injured muscles was associated with a constant activation of JAK1/2-STAT3 signaling pathway in muscles developing NHO (Alexander et al., 2019). Conversely, NHO development was attenuated in OSM receptor deficient mice (Torossian et al., 2017) or after inhibition of JAK1/2-STAT3 signaling with the small JAK1/2 tyrosine kinase inhibitor ruxolitinib (Alexander et al., 2019). Likewise, over secretion of TGF-β1 by myeloid cells via CD47 in response to extended body burns has been shown to promote burn-induced HO development (Wang et al., 2018). The increased prevalence of NHO in SCI/TBI patients with infections or concomitant inflammation (Hendricks et al., 2007; Citak et al., 2012; Reznik et al., 2014) as well as the occurrence of peri-articular HO in mechanically ventilated and immobilized severe cases of COVID-19 further support the crucial role of inflammation in this process (Meyer et al., 2020; de l’Escalopier et al., 2021; Stoira et al., 2021).

**FIGURE 2:**
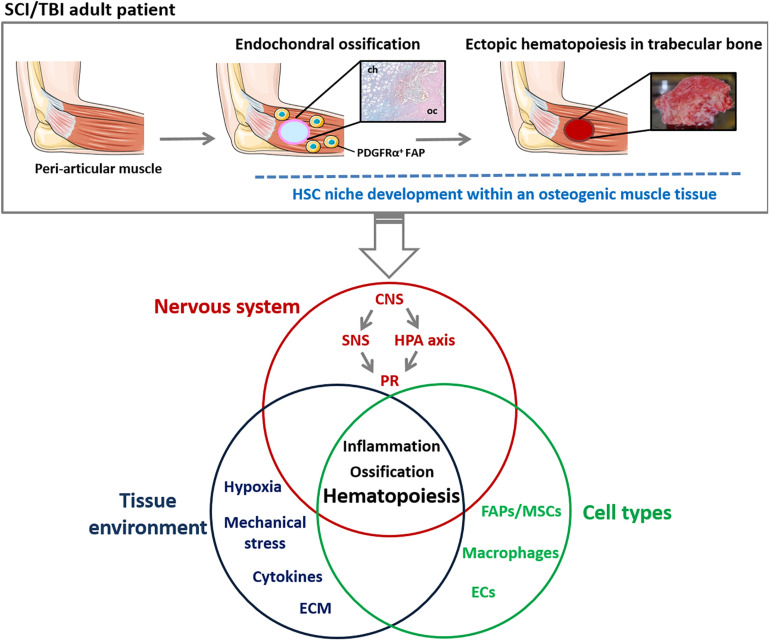
Schematic representation of mechanisms involved in ectopic hematopoietic bone development in NHO pathology. NHOs are the results of an endochondral ossification within peri-articular muscles of TBI/SCI adult patients. NHOs are characterized by the formation of a trabecular bone tissue housing HSC niches. A wide range of regulatory mechanisms are suggested to be involved in NHO pathogenesis including altered neuronal control, inflammation, macrophages and profound changes in muscle tissue environment. The singular localization of NHOs suggests a role of muscle-resident mesenchymal cells such as PDGFRα^+^ FAPs and bone metabolism in the development of ectopic hematopoiesis in adults. CNS, central nervous system; ECM, extra-cellular matrix; ECs, endothelial cells; FAP, fibro-adipogenic progenitor; HPA, hypothalamic-pituitary-adrenal; HSC, Hematopoietic stem cells; MSC, mesenchymal stromal cells; NHO, neurogenic heterotopic ossification; PR, peripheral response; SCI, spinal cord injury; SNS, sympathetic nervous system; TBI, traumatic brain injury.

Overall, these data provide a mechanistic link between persistent inflammation driving FAPs into an osteogenic fate and NHO development. Interestingly, histological sections of these ectopic bones reveal the presence of hematopoietic cells suggesting causality between abnormal FAP activation, inflammation and hematopoiesis within an osteogenic muscle microenvironment (Figure 2).

### CNS Lesion and NHO Development

Both peripheral (denervation) and central (SCI) neurologic lesions have an effect on FAPs, inducing STAT3 activation, high IL-6 secretory activity and abnormal proliferation (Madaro et al., 2018). Furthermore, CNS lesions deregulate the neuro-endocrine system causing the abnormal systemic release of a number of mediators that may trigger NHO development such as substance P (SP) or TGF-β (Genêt et al., 2015). High CNS lesions can also cause autonomic dysreflexia (AD), a life-threatening complication caused by the loss of the central control of the post-ganglionic sympathetic flow below the SCI. In retrospective studies, AD has been associated with higher prevalence of NHO in SCI and TBI patients (Hendricks et al., 2007; van Kampen et al., 2011; Putz et al., 2014). AD causes a major physiological challenge with high norepinephrine release, extreme hypertension combined with bradycardia. Whether any of the systemic drivers of AD lead to NHO development remain unexplored (Alexander et al., 2020). Severe CNS trauma also profoundly changes muscle spasticity and physical environment. The resulting mechanical stress and downstream mechano-transduction signals could facilitate NHO development because of their known effect on MSC osteogenic fate (Frith et al., 2018; Sun et al., 2018). Evidence of an abnormal activation of mechano-transductive effectors Rho/ROCK and YAP1 has recently been reported in a FOP model (Stanley et al., 2019). More interestingly, Daud et al. (1993) have shown that delayed start of passive movements to paralyzed limbs in SCI patients correlates with increased NHO occurrence.

## NHO, a Favorable Environment for the Development of Hematopoietic Stem Cell Niches in Adults

The presence of ectopic hematopoietic bones developing in muscles following CNS injuries is puzzling for the hematologist since, in adults, hematopoiesis is physiologically restricted to the BM of skeletal bones.

During fetal development, blood formation occurs in discrete anatomical extraembryonic and intraembryonic niches, generating different hematopoietic cell (HC) types (Dzierzak and Speck, 2008; Waas and Maillard, 2017). First HCs emerge in the yolk sac (YS) and generate primitive erythroblasts, macrophages and megakaryocytes. A second wave of erythro-myeloid progenitors also derived from the YS gives rise to definitive erythroid, megakaryocyte, myeloid, and multipotent progenitors initiating fetal liver (FL) hematopoiesis (Luckett, 1978; Silver and Palis, 1997). Bona fide definitive HSCs emerge by budding from specialized ECs, known as hemogenic endothelium, in the dorsal aorta, vitelline and umbilical arteries (Oberlin et al., 2002, 2010; Boisset et al., 2010). These definitive HSCs then migrate to the FL where they undergo significant proliferation (Rybtsov et al., 2016; Zhang et al., 2019) and finally reach the fetal bones and especially the BM, their life-long residence, where they become predominantly quiescent in adults (Bowie et al., 2007; Copley and Eaves, 2013). In adults, BM HSCs act as a reserve for the blood system, remaining dormant for months or years, and yet can rapidly proliferate when needed following inflammatory or cytotoxic/radiotoxic challenges (Wilson et al., 2008; Batsivari et al., 2020). Apart from some pathological situations, the BM will remain the only hematopoietic tissue in healthy adults.

### HSC Niches and Their Main Players Along Development and in Adult BM

Schofield first used the term “niche” to describe a putative HSC-specific environment in the BM that “preserved the reconstituting ability of stem cells” (Schofield, 1978). Successive specialized niches were identified during development, from the emergence of functional HSCs in the dorsal aorta, amplification in the FL and homeostasis in the adult BM (Gao et al., 2018; Daniel et al., 2020). The formation of the BM niche during fetal development has not been fully investigated, and the impact of specific niche components on fetal BM HSC phenotype, proliferation and function has still to be deciphered. In the adult, the precise nature of BM HSC niches has long been debated (Morrison and Scadden, 2014). The presence of two anatomically different niches was initially suggested: the central and the endosteal niches. Such a distinction is more and more challenged since HSCs tend to be more frequent in perivascular areas of the BM, in close proximity to ECs and perivascular stromal cells (Kokkaliaris et al., 2020) that are particularly numerous in the endosteal region (Nombela-Arrieta et al., 2013).

Within these niches, HSCs are maintained quiescent by a complex molecular interplay between cells from mesenchymal origin, ECs, neuronal cells and HSC progenies, such as megakaryocytes and macrophages. Diffusible factors including inflammatory cytokines and extra-cellular matrix components perfect this molecular network, subtly controlling the fate of HSCs (Méndez-Ferrer et al., 2020).

Apart from stromal cells, macrophages are essential to HSC regulation within niches. They are the most abundant HCs in the dorsal aorta when the number of intra-aortic hematopoietic cluster peaks, and are suggested to promote definitive HSC formation from the dorsal aorta hemogenic endothelium through pro-inflammatory signaling cascades (Yokomizo and Dzierzak, 2010). Among these pro-inflammatory signals (Luis et al., 2016; Hayashi et al., 2019), tumor necrosis factor (TNF) (Espín-Palazón et al., 2014), interferons (IFN) (Sawamiphak et al., 2014), IL-1 (Orelio et al., 2009), and OSM (Miyajima et al., 2000) play a major role in the regulation of embryonic and fetal hematopoiesis. Macrophages are also initiators of the endothelial-to-hematopoietic transition since hemogenic ECs that receive these cues, undergo endothelial-hematopoietic transition and form HSCs (Mariani et al., 2019). In adult hematopoiesis, macrophages exert several other functions in the BM niches. They participate in the retention of HSCs through their interactions with MSCs and possibly ECs and the modulation of the expression of proteins such as CXCL12 (Winkler et al., 2010), VCAM-1 and KIT ligand. Furthermore, supraphysiological expansion of the monocyte/macrophage compartment by prolonged administration of a stable recombinant form of macrophage colony-stimulating factor (CSF-1) expand the HSC compartment in the BM (Kaur et al., 2021). Reciprocally, BM-resident macrophages are necessary to reconstitute HSC niches after lethal irradiation and support HSC engraftment (Kaur et al., 2017, 2018). The stromal expression of CXCL12 follows a circadian regulation that is under the control of sympathetic nerve fibers, connecting macrophages and the nervous system in the regulation of HSC trafficking (Katayama et al., 2006; Méndez-Ferrer et al., 2020). In the context of infections or inflammatory stresses, macrophages and ECs produce G-CSF that participates in the mobilization of HSCs from the BM into the circulation while promoting myelopoiesis. G-CSF also contributes in suppressing osteoblast function by directly down-regulating CXCL12 expression in the endosteal niche or by indirect mechanisms including signals from the sympathetic nervous system (SNS) (Winkler et al., 2010; Christopher et al., 2011) (for review: Mitroulis et al., 2020).

### Neuronal and Neuroendocrine Regulation of Bone and HSC Niche

In the context of NHO, it is noteworthy to integrate the role of the nervous system as an important regulator of bone remodeling and hematopoiesis homeostasis. Since the discovery of skeleton innervation by Calvo (1968), other groups including that of Paul Frenette have further explored from this pioneering observation and described the neuronal regulation of bone and BM (see for review: Maryanovich et al., 2018). In addition to a mineral constituent regulation, bone homeostasis is controlled by long-range signals such as leptin, glucocorticoids and parathyroid hormone produced by the adipose tissue, the adrenal glands, and the parathyroid glands, respectively, and by signals originating from the nervous system.

Besides its role in energy homeostasis, leptin plays a major role in neuroendocrine regulation and bone metabolism. The expression of leptin receptor on adult MSCs, osteoblasts and chondrocytes, suggests direct effects on bone growth and metabolism. Leptin can also indirectly modulate bone formation through effectors downstream of the hypothalamus such as estrogen, cortisol, IGF-1 and parathyroid hormone, and through activation of local adrenergic signaling at the osteoblast level via β2 adrenergic receptors (AR) (Upadhyay et al., 2015; Wang et al., 2020 for review). Leptin also inhibits the neuronal activity of serotonergic neurons and decrease brain-derived serotonin synthesis (see for review: Maryanovich et al., 2018; Karsenty, 2020). Sensory and sympathetic nerves also participate in bone homeostasis through neurotransmitters including nerve growth factor (NGF), calcitonin gene-related peptide (CGRP), SP, and semaphorin 3A and through norepinephrine/noradrenaline released by the SNS (Wang et al., 2020).

As reported above, HSCs are mainly located in perivascular areas of the adult BM, comprising both sinusoidal and arteriolar blood vessels. The arteriolar structures are highly innervated by SNS fibers. The neuroreticular complex formed by SNS nerves and perivascular MSCs has been reported to be a central regulator of HSC quiescence within BM niches (see for review: Maryanovich et al., 2018). Interestingly, a variable proportion of these perivascular stromal cells expressed neural-related markers such as LepR, NG2, and Nestin.

The close relationship between CNS, SNS, Parasympathetic Nervous System, bone metabolism and HSC migration, differentiation and self-renewal is illustrated by the important role of the circadian norepinephrine release by SNS nerves which triggers β3 AR and β2 AR expressed by BM mesenchymal cells and osteoblasts in both humans and mice (Golan et al., 2018, 2019). Cholinergic signaling in the BM via neurons from both the PNS and SNS in tandem with adrenergic signaling derived from the SNS is also reported to contribute to regulate the circadian regulation of CXCL12 expression in the BM (García-García et al., 2019; García-García and Méndez-Ferrer, 2020). Likewise, the CNS exerts long range regulation of HSCs. For instance, muscarinic type 1 acetylcholinergic receptors in the brain regulate HSC mobilizing response by stimulating the hypothalamic-pituitary-adrenal (HPA) axis and glucocorticoid secretion (Pierce et al., 2017). Similarly, afferent sensory nociceptive nerves in the BM have been found to regulate HSC expansion, differentiation and mobilization in concert with SNS nerves via CGRP release (Gao et al., 2020).

Beside its role in adult stem cell niche homeostasis, the neural system is also an early regulator of the embryonic niches when stem cells are specified. Apart from a direct innervation of the HSC niche, recent findings show different modes of neural control, including systemic delivery of CNS-derived hormones, locally by neural crest-derived MSCs, and intrinsically by HCs expressing neural receptors and neurotransmitters (Fitch et al., 2012; Damm and Clements, 2017). During development, hypoxia-induced neuronal synthesis of serotonin is a key process for embryonic HSC production in the aorta region. Neuronal serotonin activates the HPA axis and glucocorticoid receptor activity, which in turn, induces HSC production (Kwan et al., 2016). Although hypoxia was the only stress-inducing stimulus tested, it is conceivable that other common stresses that the embryo experiences such as temperature, metabolic, or oxidative stress would also promote blood cell formation through the HPA axis. Such mechanisms were also described in adults, where muscarinic acetylcholine receptors in the brain regulate HPA axis and glucocorticoid release by the adrenal glands that impact HSC trafficking (Pierce et al., 2017).

### NHO: An Osteogenic Muscle Tissue That Houses HSC Niches!

Addressing whether HOs form bona fide HSC niches is challenging since these ectopic bone tissues develop in inflamed muscles following severe neurological lesions. Few groups have reported the presence of marrow-like tissue in HOs. Histological descriptions are reported after abdominal surgery (Wang et al., 2004; Christofi et al., 2008), aortic valve graft (Lis et al., 2009; Singh and Fleshman, 2011) or cervical spine meniscoid (Farrell et al., 2017). Furthermore, clonogenic hematopoietic progenitors associated with histologically-defined stromal cells have been described in HOs from severe combat-injured orthopedic patients (Davis et al., 2013). However, the evidence for functional HSC niches comes from studies in SCI and TBI patients (Torossian et al., 2017). These studies identified phenotypic CD34^+^ hematopoietic stem/progenitor cells in NHO marrows. Depending on patients, their level was equivalent or slightly lower than in the healthy BM. Some of those CD34^+^ cells were quiescent, expressed a side-population phenotype (Goodell, 2005) and were capable of long-term human hematopoietic reconstitution when transplanted into immunodeficient mice, thus meeting the functional definition of HSCs. NHO marrow also contained functional CD45^–^CD34^–^CD73^+^CD90^+^CD105^+^ MSCs able to differentiate in osteoblasts, adipocytes and chondrocytes and to support long-term human hematopoiesis in culture. More importantly, when seeded on hydroxyapatite scaffolds and implanted into nude mice, NHO-derived MSCs created a supportive osteogenic microenvironment for murine hematopoiesis (Torossian et al., 2017). In agreement with these results, their transcriptomic signature showed a molecular network required for HSC support. Intriguingly, this signature was associated with a neuronal imprinting, arguing in favor of the brain-bone-blood triad concept proposed by Lapidot (Lapidot and Kollet, 2010). ECs and their progenitors could also be isolated from NHO marrow according to their CD45^–^CD31^+^CD144^+^CD34^+^ phenotype. They were functional as demonstrated by colony formation on plastic, expansion in culture as a cobblestone monolayer, vascular network development in matrigel and overexpression of VCAM-1 and ICAM-1 after TNFα stimulation (Torossian et al., 2017).

By demonstrating that NHOs contain a marrow tissue in which HSCs can proliferate and differentiate within a suitable and functional osteogenic/mesenchymal and vascular microenvironment, these studies acknowledge that NHO ectopic bones house HSC niches. It is noteworthy that, in NHO patients, the altered neuronal control most likely contributes to the generation of hematopoietic bones comparable to the BM, in muscles. More importantly, the NHO paradigm emphasizes the role of muscle microenvironment and inflammation in their development (Figure 2).

Intriguingly, while the role of central, sympathetic and parasympathetic nervous systems in regulating hematopoiesis in the BM of skeletal bones is well described, it is not known whether the hematopoietic BM of NHOs is actually innervated, and if so, what roles these nerves would play in establishing, maintaining and regulating hematopoiesis in the NHO marrow.

### Lessons From NHO for a Better Understanding of HSC Development

Independent studies of vertebrate hematopoietic development (Waas and Maillard, 2017; Dzierzak and Bigas, 2018) and NHO pathogenesis (Davis et al., 2013; Torossian et al., 2017) reveal that embryonic and adult NHO HSCs share similarities. Both develop in soft tissues within niches under the control of regulatory mechanisms including macrophages, inflammation, and the nervous system. However, significant changes in the composition of the HSC pool, as well as in their cell cycling properties and repopulating abilities are observed between the fetal hematopoietic tissues and the adult BM (Copley and Eaves, 2013; Mirshekar-Syahkal et al., 2014).

In Osterix-null (Osx^–/–^) mice that lack osteoblasts and osteolineage cells, the vasculature within the nascent bones and bone marrow can sustain multilineage proliferative progenitors but not long-term HSCs. As a result, wild-type HSC transplanted in Osx embryos engraft the liver but not the nascent BM. Therefore, interactions with osteoblasts within fetal bone regulate HSC quiescence and homing ability (Coşkun et al., 2014). In the adult BM, the role of osteolineage cells is more questionable since the deletion of *Cxcl12* or *Kitl* gene from Osx^+^ osteoprogenitors has more effect on hematopoietic progenitors than on proper HSCs (Ding and Morrison, 2013). In contrast, both genes need to be expressed in ECs and in immature *Lepr*^+^ MSCs (that form osteoprogenitors) for HSCs to be maintained (Greenbaum et al., 2013). These results emphasize the importance of osteolineage cells, and most likely other cells from mesenchymal origin, in establishing and sustaining HSC phenotype, cell cycling balance and function during development and adult life.

Understanding the development of hematopoiesis in an adult osteogenic muscle environment as observed in NHO could help gain further insights on the role of bone forming cells in this process. Identification of stage-specific factors that orientate HSC developmental state during fetal bone development must be harnessed to gain more mechanistic insights into NHO development. Similarly, understanding the cellular origin of NHO, the role of inflammation and muscle environment might contribute to a better understanding of the impact of specific niche components on fetal BM HSC properties.

## Conclusion

In the recent few years, knowledge about NHO pathogenesis has been considerably improved as accredited by the rapidly increasing number of publications in the field. These progresses were mainly due to the development of more suitable animal models and to the availability of patient samples thanks to well organized cohorts. The current review focusing on the hematopoietic features of NHO ossifications, attempts to recapitulate how a favorable environment for the development of bone with HSC niches can develop in adult muscles following central neurological lesions. It emphasizes the role of a persistent inflamed muscle environment driving FAPs to an osteogenic fate initiating the development of ossification followed by the establishment of a mature hematopoietic bone tissue.

However, there are still numerous questions in respect to the molecular mechanisms underlying this complex and multifactorial pathological process. Among those, the potential differences between normal endochondral ossification and neurogenic HO in terms of signaling events, cell type involvement and environment remains unanswered. Likewise, how an inflamed adult muscle environment becomes pro-osteogenic and thereafter hematopoietic, and what is the influence of altered nervous and neuroendocrine systems as well as hypoxia in this process? Does the neoformation of hematopoietic bones in muscles mimic what happens during development and can we learn from NHO for a better identification of stage-specific factors that specify HSC developmental state during fetal bone development? Is the impaired mobility of patients a trigger in the development of NHO and does an early and adequate mobilization of patients can avoid or at least reduce its evolution?

Gathering surgeons, clinicians, specialists in physical medicine/rehabilitation and researchers within a European/International consortium would be a provocative initiative for developing translational collaborative projects to better understand NHO pathogenesis and, armed with this knowledge, enable the identification of new targets to treat and if possible prevent NHO development. Moreover, such knowledge may also provide new insights for cell therapy needs and for improving treatment of blood and bone disorders.

## Author Contributions

DG, FT, EO, KA, JG, H-WT, MS, J-PL, M-CL, and SB wrote the manuscript. DG, EO, M-CL, and SB prepared the figures. All authors revised the manuscript and approved the submitted version.

## Conflict of Interest

The authors declare that the research was conducted in the absence of any commercial or financial relationships that could be construed as a potential conflict of interest.
